# Response: Commentary: Supplier-dependent differences in intermittent voluntary alcohol intake and response to naltrexone in Wistar rats

**DOI:** 10.3389/fnins.2016.00442

**Published:** 2016-09-30

**Authors:** Lova Segerström, Erika Roman

**Affiliations:** Research Group Neuropharmacology, Addiction and Behavior, Department of Pharmaceutical Biosciences, Uppsala UniversityUppsala, Sweden

**Keywords:** animal model, behavior, phenomics, phenotypes, addiction, alcohol use disorders, Y-maze, open field

The authors appreciate Prof. Kalueffs interest in our work (Momeni et al., 2015). The issues highlighted (Kalueff, 2016) are indeed of great importance. Many factors associated with animal husbandry can potentially have an impact on behavior (Castelhano-Carlos and Baumans, 2009), and a more thorough description of experimental conditions is often warranted for replicability and/or comparison across labs. Indeed, attempts to compare results across labs through careful standardization show that the lab environment, including experimenter handling, give rise to variation (Crabbe et al., 1999; Wahlsten et al., 2003; Riedel and Spruijt, 2016); also observable in experiments with automated home-cage based tracking equipment with minimal experimenter handling (Riedel and Spruijt, 2016). An alternative approach of reduced standardization has therefore been suggested (Voelkl and Würbel, 2016).

Momeni et al. (2015) attempted to harness of some of the known differences in Wistar rats from different suppliers (Palm et al., 2011a,b, 2012; Goepfrich et al., 2013) in order to create a more heterogeneous group of animals. Kalueff (2016) suggests that the differences observed by Momeni et al. (2015) are due to environmental factors and/or in combination with acclimatization. These are important aspects, often poorly described in publications, but in our opinion not the full explanation behind the results (Momeni et al., 2015).

Animal handling and husbandry vary, see Supplementary Table and Langer et al. (2011) for additional information, which may impact not only our results. The most striking observation (Supplementary Table) is that Wistar rats and CD-1 mice are kept in the same housing room at Charles River GmbH (Personal communication, Charles River distributor, Scanbur employee), a particular concern to users of CD-1 mice from that supplier. A comparison between Supplementary Table and Kalueff (2016) render discrepancies in information, i.e., number of animals per cage. This may be referred to EU Guidelines, stating that body weight and not age determine the number of animals per cage, applicable to Momeni et al. (2015). Notably, the number of animals allowed per cage in different cage types varies between EU, UK and USA Guidelines, respectively (Tecniplast, 2016).

The impact of transportation procedures (Supplementary Table) should not be underestimated. The distance transported *per se* (Kalueff, 2016) is likely of less importance with regard to the animals used in Momeni et al. (2015), considering that negligible effects were demonstrated in mice bred locally vs. shipped (Wahlsten et al., 2003). Moreover, the undisturbed acclimatization period of 2 weeks upon arrival is in line with recommendations for male rats (Arts et al., 2012, 2014a,b), also when transferred from normal to reversed light/dark cycle (Arts et al., 2016).

An additional factor of potentially large importance for behavioral studies worth emphasizing is the light/dark cycle. Some publications lack specific information about photoperiod for behavioral testing. Behavioral testing of nocturnal animals should preferably be conducted during the dark (active) phase, unless it is established that the behavioral measures collected are not impacted by photoperiod (Castelhano-Carlos and Baumans, 2009; Hawkins and Golledge, 2016). Moreover, the introduction of individually ventilated cage (IVC) systems may impact on animal physiology (Castelhano-Carlos and Baumans, 2009), and result in behavioral differences when IVC-housed animals are compared to animals housed in conventional open cage systems (Kallnik et al., 2007; Mineur and Crusio, 2009; Logge et al., 2013).

Despite acknowledging mentioned differences in animal handling and husbandry, additional factors may be of importance for the marked supplier-dependent differences between Wistar rats (Langer et al., 2011; Palm et al., 2011a,b, 2012; Goepfrich et al., 2013; Momeni et al., 2015; Theilmann et al., 2016; Wood et al., 2016). Wood et al. (2016) presents a simplified scheme of derivation of commercially available Wistar rats. Recently, a single point mutation in exon 3 of the *Grm2* gene was reported, resulting in a premature stop codon of the metabotropic glutamate 2 (mGlu2) receptor, and concomitant loss of functional protein expression. This mutation has a widespread prevalence in Wistar rats, particularly those known as Han Wistar (Wood et al., 2016). The possible prevalence of other mutations in Wistar rats from different suppliers remains to be investigated.

In Momeni et al. (2015) the aim was indeed to apply a behavioral phenomics approach (Gerlai, 2002; Kalueff, 2016) to create a heterogenic group by combining Wistar rats from different suppliers; known to display different characteristics and thus pose as a representative of the heterogeneity in a human population (Stewart and Kalueff, 2015) to explore how subgroups with different behavioral characteristics acquire and maintain voluntary alcohol intake. We were hoping to find a pattern similar to that described by Kalueff (2016). However, analysis of behavioral data (open field and Y-maze) and voluntary alcohol intake revealed differences and concomitant skew subgroup formations, primarily in open field performance and voluntary alcohol intake, that were of supplier-dependent origin rather than equally distributed in the heterogeneous group (Table 1 in Momeni et al., 2015). Kalueff (2016) applied a Spearman correlation analysis to the summary subgroup data obtained from Table 1 in Momeni et al. (2015), i.e., the distribution of the heterogeneous cohort of animals into different subgroups based on behavior and voluntary alcohol intake, and presented correlations that imply possible “strain-specific differences in the higher-order phenotypes,” while also acknowledging that more accurate results may be generated by analyzing raw data (Kalueff, 2016). Due to the skew subgroup formations in Momeni et al. (2015), such correlations were originally not considered relevant to perform. Indeed, Spearman rank order correlations on selected raw data presented in Momeni et al. (2015) reveal no correlations (Table 1) in support of the preliminary results presented by Kalueff (2016).

**Table 1:** **Correlation matrix comprising selected parameters from the open field (OF) and Y-maze (YM) tests, and voluntary alcohol intake during weeks one and six of access (see Momeni et al., 2015 for details)**.

	**Duration (%) inner zone OF**				**Total activity OF**				**Distance (cm) OF**				**CA (%) YM**				**Total alternations YM**				**Distance (cm) YM**				**Alcohol intake (g/kg) w1**			
	**All**	**Rcc**	**Crl**	**Tac**	**All**	**Rcc**	**Crl**	**Tac**	**All**	**Rcc**	**Crl**	**Tac**	**All**	**Rcc**	**Crl**	**Tac**	**All**	**Rcc**	**Crl**	**Tac**	**All**	**Rcc**	**Crl**	**Tac**	**All**	**Rcc**	**Crl**	**Tac**
Duration (%) inner zone OF	−	−	−	−																								
Total activity OF	0.92	0.90	0.82	0.84	−	−	−	−	−																			
Distance (cm) OF	0.69	0.64	n.s.	0.58	0.74	0.63	0.66	0.62	−	−	−	−																
CA (%) YM	n.s.	0.49	n.s.	n.s.	n.s.	n.s.	n.s.	n.s.	n.s.	n.s.	n.s.	n.s.	−	−	−	−												
Total alternations YM	0.42	n.s.	n.s.	n.s.	0.49	n.s.	n.s.	n.s.	0.52	n.s.	n.s.	n.s.	n.s.	n.s.	n.s.	n.s.	−	−	−	−								
Distance (cm) YM	0.44	n.s.	n.s.	n.s.	0.49	n.s.	n.s.	n.s.	0.56	n.s.	n.s.	n.s.	n.s.	n.s.	n.s.	n.s.	0.92	0.85	0.72	0.96	−	−	−	−				
Alcohol intake (g/kg) w1	−0.30	n.s.	n.s.	n.s.	−0.26	n.s.	n.s.	n.s.	−0.29	n.s.	n.s.	n.s.	n.s.	n.s.	n.s.	n.s.	n.s.	n.s.	0.76	n.s.	n.s.	n.s.	0.79	n.s.	−	−	−	−
Alcohol intake (g/kg) w6	n.s.	n.s.	n.s.	n.s.	n.s.	n.s.	n.s.	n.s.	n.s.	n.s.	n.s.	n.s.	n.s.	n.s.	n.s.	n.s.	n.s.	n.s.	n.s.	n.s.	n.s.	n.s.	n.s.	n.s.	n.s.	0.66	n.s.	n.s.

*Data are presented as Spearman rank order correlation ρ-values with a statistical significance at p < 0.05*.

*CA, correct alternations in the Y-maze; n.s., not statistically significant; OF, open field; w, week; YM, Y-maze*.

The analysis of open field data in all rats, i.e., the heterogeneous group, revealed a positive correlation between time spent in the inner zone (interpreted as risk-taking behavior) and total activity; an effect also present within the respective group of rats (Table 1). This finding was reported in Momeni et al. (2015) and is in agreement with previous findings (Momeni et al., 2014). Moreover, measures of general activity (distance moved, total activity and total alternations) in the open field and Y-maze tests were correlated in the heterogeneous group of all rats, while only correlations within the respective tests were found in the Rcc, Crl, and Tac groups, respectively (Table 1). A novel finding was that in the heterogeneous group (all rats), total activity, distance moved, and the time spent in the inner zone of the open field was negatively correlated to voluntary alcohol intake during the first week of access, i.e., at acquisition. These correlations were weak, driven predominantly by Crl rats (Supplementary Figure), as they are characterized by longer duration in the inner zone and higher total activity compared to Rcc and Tac rats (Momeni et al., 2015), and absent in the respective group of Rcc, Crl, or Tac rats (Table 1). In agreement with our previous finding (Momeni et al., 2014) and proof of robustness, no association between risk-taking behavior and voluntary alcohol intake was revealed in Rcc rats. To summarize, the complementary correlational analyses performed herein do not support the preliminary findings presented by Kalueff (2016).

In conclusion, the important questions raised by Kalueff (2016), to some extent expanded upon herein, can hopefully raise the attention to the many factors to consider when performing animal experiments in general, and studies in the field of behavioral phenomics and neuroscience in particular. This is all in a strive toward more robust behavioral research with the aim of developing experimental tests and models with high population validity that better parallel findings in humans.

## Author contributions

Study PI: ER. Response outline: LS and ER. Analyzed the data: ER. Drafted the manuscript: ER. Revised and approved the final version: LS and ER.

### Conflict of interest statement

The authors declare that the research was conducted in the absence of any commercial or financial relationships that could be construed as a potential conflict of interest.
