# Supplementary material for: Response: Commentary: Supplier-dependent differences in intermittent voluntary alcohol intake and response to naltrexone in Wistar rats
Source: Front Neurosci. 2016 Sep 30;10:442. doi: 10.3389/fnins.2016.00442 (PMC5042965; doi:10.3389/fnins.2016.00442)
Supplement: Supplementary file 1 [file Table1.pdf]

Response: Commentary: Supplier-dependent differences in intermittent voluntary alcohol intake and response to naltrexone in Wistar rats.

Lova Segerström, Erika Roman*

Supplementary Table. Brief background information regarding housing and transportation of the Wistar rats obtained from the suppliers Charles River GmbH (Crl:WI), Taconic Farms A/S (HanTac:WH) and Harlan Laboratories B.V. (now Envigo; RccHan™:WI) used in Momeni et al (2015). Data were collected by personal communication with the respective supplier, and complement that obtained by others (Kalueff, 2016; Langer et al., 2011). Note that Charles River GmbH report housing rats and mice in the same room.

	Charles River GmbH	Taconic Farms A/S	Harlan Laboratories B.V. (now Envigo)
**Weaning age**	3 weeks of age	Approximately 20 days of age	21-28 days of age
**Housing**			
Cage type	Type III high or Type IV	1500U IV S	RC1 cages
Cage dimensions (width x length x height)	42 x 26 x 18 cm (Type III high), 59 x 38 x 20 cm (Type IV)	48 x 35.5 x 21 cm	38 x 51 x 17 cm
Cage color	Transparent	Opaque	Opaque/transparent
Cage enrichment	Paper/Nage wood	Wood blocks, nesting material	Plastic or cardboard tunnels, wood blocks
Group housing	Yes	Yes	Yes
**Holding rooms**			
Light/dark cycle	Normal, 12/12 h	Normal, 12/12 h	Normal, 12/12 h
Males and females housed in the same room	Yes	Yes	Yes
Rats and mice housed in the same room	Yes, Wistar rats and CD-1 mice	No	No
**Transportation**			
Packing at vendor/Arrival in Uppsala, Sweden	Monday/Tuesday	Tuesday/Thursday	Monday/Wednesday
Mode of transportation	Car	Car	Car

References

Kalueff, A.V. (2016). Commentary: Supplier-dependent differences in intermittent voluntary alcohol intake and response to naltrexone in Wistar rats. *Front Neurosci* 10**,** 82.

Langer, M., Brandt, C., and Loscher, W. (2011). Marked strain and substrain differences in induction of status epilepticus and subsequent development of neurodegeneration, epilepsy, and behavioral alterations in rats. [corrected]. *Epilepsy Res* 96**,** 207-24.

Momeni, S., Segerström, L., and Roman, E. (2015). Supplier-dependent differences in intermittent voluntary alcohol intake and response to naltrexone in Wistar rats. *Front Neurosci* 9**,** 424.
