# Supplementary material for: Response: Commentary: Supplier-dependent differences in intermittent voluntary alcohol intake and response to naltrexone in Wistar rats
Source: Front Neurosci. 2016 Sep 30;10:442. doi: 10.3389/fnins.2016.00442 (PMC5042965; doi:10.3389/fnins.2016.00442)
Supplement: Supplementary file 2 [file Image1.pdf]

Response: Commentary: Supplier-dependent differences in intermittent voluntary alcohol intake and response to naltrexone in Wistar rats.

Lova Segerström, Erika Roman*

Supplementary Figure.

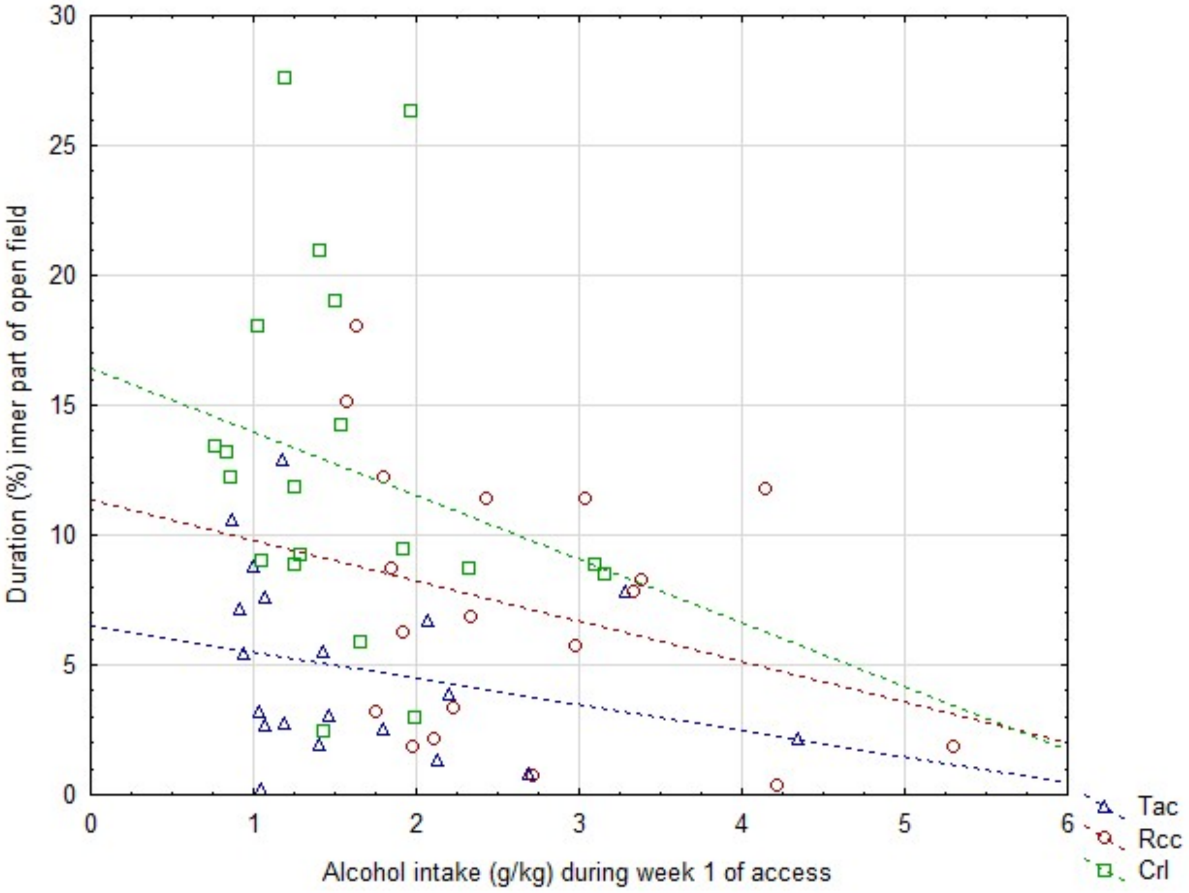

Correlations between time spent (%) in the inner zone of the open field test and voluntary alcohol intake during the first week of access in Rcc (red circles), Crl (green squares) and Tac (blue triangles) Wistar rats, for details see Momeni et al. (2015). No significant correlation was found within the respective supplier (Table 1). Data are presented as Spearman rank order correlations (Statistica 12.0, StatSoft Inc., Tulsa, OK).

References

Kalueff, A.V. (2016). Commentary: Supplier-dependent differences in intermittent voluntary alcohol intake and response to naltrexone in Wistar rats. *Front Neurosci* 10, 82.

Momeni, S., Segerström, L., and Roman, E. (2015). Supplier-dependent differences in intermittent voluntary alcohol intake and response to naltrexone in Wistar rats. *Front Neurosci* 9, 424.
